# Hemoglobin Promotes *Staphylococcus aureus* Nasal Colonization

**DOI:** 10.1371/journal.ppat.1002104

**Published:** 2011-07-07

**Authors:** Melissa Pynnonen, Rachel E. Stephenson, Kelly Schwartz, Margarita Hernandez, Blaise R. Boles

**Affiliations:** 1 Department of Otolaryngology-Head and Neck Surgery, University of Michigan, Ann Arbor, Michigan, United States of America; 2 Department of Molecular, Cellular, and Developmental Biology, University of Michigan, Ann Arbor, Michigan, United States of America; National Institute of Allergy and Infectious Diseases, National Institutes of Health, United States of America

## Abstract

*Staphylococcus aureus* nasal colonization is an important risk factor for community and nosocomial infection. Despite the importance of *S. aureus* to human health, molecular mechanisms and host factors influencing nasal colonization are not well understood. To identify host factors contributing to nasal colonization, we collected human nasal secretions and analyzed their ability to promote *S. aureus* surface colonization. Some individuals produced secretions possessing the ability to significantly promote *S. aureus* surface colonization. Nasal secretions pretreated with protease no longer promoted *S. aureus* surface colonization, suggesting the involvement of protein factors. The major protein components of secretions were identified and subsequent analysis revealed that hemoglobin possessed the ability to promote *S. aureus* surface colonization. Immunoprecipitation of hemoglobin from nasal secretions resulted in reduced *S. aureus* surface colonization. Furthermore, exogenously added hemoglobin significantly decreased the inoculum necessary for nasal colonization in a rodent model. Finally, we found that hemoglobin prevented expression of the *agr* quorum sensing system and that aberrant constitutive expression of the *agr* effector molecule, RNAIII, resulted in reduced nasal colonization of *S. aureus*. Collectively our results suggest that the presence of hemoglobin in nasal secretions contributes to *S. aureus* nasal colonization.

## Author Summary

*Staphylococcus aureus* is a medically important human pathogen that is found in the nasal passages of approximately 1/3 of the population. The nose serves as a reservoir for spread of this pathogen and predisposes the host to potential infection. Factors contributing to *S. aureus* nasal colonization are only beginning to be elucidated. The collection and analysis of human nasal secretions provided evidence that the presence of hemoglobin in nasal secretions can promote *S. aureus* nasal colonization. Hemoglobin reduced expression of the *S. aureus agr* quorum sensing regulatory system known to be involved in surface colonization, and it was found that induction of the *agr* system reduced nasal colonization. These findings suggest that individuals experiencing frequent nosebleeds would be prone to *S. aureus* colonization and epidemiological data supports these findings. By understanding host factors and bacterial molecular mechanisms involved in nasal colonization we may one day be able to design novel decolonization strategies.

## Introduction

*Staphylococcus aureus* is a human commensal and the causative agent of many serious acute and chronic infections [1]. The primary reservoir for *S. aureus* is the nasal cavity [2], [3]. Asymptomatic colonization occurs in approximately 20% of the normal population, another 60% are transiently colonized and the remaining 20% appear to be rarely or never colonized [4], [5]. Why some individuals are prone to colonization while others resist colonization is not understood. *S. aureus* nasal colonization is a known risk factor for several infections including bacteremia [6], [7], postoperative infections [8], and diabetic foot ulcer infections [9]. Treatment with the topical antibiotic mupirocin has proven to be effective at reducing nasal colonization and the risk of postoperative infection [3], [6]. However the appearance of mupirocin resistance threatens this nasal eradication strategy [10]. Therefore an improved understanding of nasal carriage is needed to foster development of new strategies to reduce colonization and subsequent infection.

*S. aureus* nasal colonization likely involves both host and bacterial determinants. Studies analyzing patterns of nasal carriage suggest that host factors may influence *S. aureus* nasal colonization [3]. Different carriage rates have been observed among different ethnic groups and families [11], [12]. Furthermore, human nasal secretions show variability in supporting growth or having antimicrobial activity against *S. aureus* [13]. Nasal host receptors may also vary among individuals as *S. aureus* adherence to desquamated epithelial cells from carriers is significantly greater than for non-carriers [14]. Bacterial products influencing colonization have also been identified. These include sortase A, teichoic acid, clumping factor B, capsule, iron-regulated surface determinant A, alkyl hydroperoxide reductase, catalase, and the autolysin SceD [15]–[21]. In addition, recent evidence has shown that polymicrobial interactions likely play a role in *S. aureus* nasal colonization [22], [23]. Taken together these studies suggest that nasal carriage is a multifactorial process that is influenced by host determinants, bacterial products, and polymicrobial interactions.

The goal of this work was to determine if components of human nasal secretions are capable of promoting *S. aureus* colonization. Human nasal secretions were collected and examined for their ability to promote *S. aureus* surface colonization. Analysis of nasal secretions that promoted *S. aureus* surface colonization revealed that hemoglobin was both necessary and sufficient for this activity. Hemoglobin promoted surface colonization in a multitude of *S. aureus* strains. Furthermore, the addition of hemoglobin to a cotton rat model reduced the inoculum size necessary to establish *S. aureus* nasal colonization. Finally, hemoglobin was found to inhibit induction of the *agr* quorum sensing system and a construct expressing the *agr* effector molecule RNAIII displayed decreased nasal colonization. Our findings suggest that nasal secretions containing hemoglobin have the ability to modulate *S. aureus* gene expression and increase nasal colonization.

## Materials and Methods

### Ethics statement

Animal work in this study was carried out in strict accordance with the recommendations in the Guide for the Care and Use of Laboratory Animals of the National Institutes of Health. The protocol was approved by the Committee on Use and Care of Animals (UCUCA) of the University of Michigan (Permit Number:10394). All efforts were made to minimize pain and discomfort during the procedure. Work involving collection of nasal secretions from human subjects was approved by the University of Michigan Institutional Review Board, approval number IRB00001996. Written informed consent was provided by study participants.

### Strains, growth conditions and reagents

The bacterial strains used in this study are described in Table 1. Strains of *Escherichia coli* were grown in Luria-Bertani broth or Luria agar plates, and growth medium was supplemented with ampicillin (100 µg/ml) or chloramphenicol (10 µg/ml) as needed for maintenance of plasmids. Except where noted, *S. aureus* strains were grown in tryptic soy broth (TSB) or tryptic soy agar (TSA). For selection of chromosomal markers or maintenance of plasmids, *S. aureus* antibiotic concentrations were (in µg/ml): erythromycin (Erm) 10; chloramphenicol (Cam) 10. All reagents were purchased from Fisher Scientific (Pittsburg, PA) or Sigma (St. Louis, MO) unless otherwise indicated. The human proteins used were purchased from Sigma at the highest available purity: hemoglobin (H7379), IgK (K3502), carbonic anhydrase (C4396), orosomucoid (G9885), IgG (I4506), albumin (A4327), hemopexin (H9291), transferrin (90190), lactoferrin (L0520), plasminogen (P7999), myoglobin (M0630), fibronectin (F2006), and collagen (C7521). Apohemoglobin was prepared using the cold acid acetone precipitation method [24].

**Table 1:** Strain and plasmid list.

Strain or plasmid	Relevant Genotype	Resistance	Source or reference
*Escherichia coli*			
DH5α-E	Cloning strain	None	Invitrogen
AH426	ER2566 *ΔgshA::cm*/pDnaB8 – AIPI	Amp	[31]
*Staphylococcus aureus*			
RN4220		None	[51]
SH1000	*agr* Type I	None	[52]
SH1001	SH1000 *Δagr::tet*	Tet	[52]
BB429	FRI1169(*agr* Type I)/pDB59	Cam	[31]
BB430	502a(*agr* Type II)/pDB59	Cam	[31]
BB431	ATCC25923(*agr* Type III)/pDB59	Cam	[31]
Newman	*agr* Type I	None	[53]
AH462	SH1000/pDB59	Cam	[27]
AH500	SH1000/pAH9	Erm	[27]
BB2041	Newman *isdB::erm*	Erm	[37]
BB2042	Newman *ΔisdH*	None	[37]
BB2043	Newman *isdB::erm*, *ΔisdH*	Erm	[37]
BB2047	BB2041/pDB59	Cam, Erm	This work
BB2044	BB2042/pDB59	Cam, Erm	This work
BB2048	BB2043/pDB59	Cam, Erm	This work
BB2146	SH1000 spectinomycin res.	Spec	This work
BB2169	BB2146/pALC2084	Cam	This work
BB2170	BB2146/pALC2073-RNAIII	Cam	This work
Plasmids			
pDB59	P_3_-GFP reporter	Amp, Cam	[33]
pAH9	*sarA* promoter P_1_-RFP	Amp, Erm	[27]
pDNAB8 - AIPI	AIP-I intein plasmid	Amp	[31]
pALC2084	Tet inducible GFP expression vector	Cam	[54]
pALC2073-RNAIII	Tet inducible RNAIII expression vector	Cam	[55]

### Recombinant DNA and genetic techniques

Restriction and modification enzymes were purchased from New England Biolabs (Beverly, MA). All DNA manipulations were performed in *E. coli* strain DH5α. Oligonucleotides were synthesized at Integrated DNA Technologies (Coralville, IA). Plasmids were transformed into *S. aureus* RN4220 by electroporation and then purified and moved to other indicated *S. aureus* strains by electroporation. Plasmid pALC2073-RNAIII was made by PCR amplification of RNAIII using primers GTTGTTGAATTCTTCATTACAAAAAAGGCCGCGAGCTTGGGA and GTTGTTGGTACCAGATCACAGAGATGTGATGGAAAATAGTTG. The PCR product was digested with KpnI and EcoRI and ligated into the pALC2084 vector that had been digested with the same restriction enzymes.

### Nasal colonization model and human nasal secretion collection

The cotton rat nasal colonization model described by Kokai-Kun was utilized in this study [25]. Briefly, *S. aureus* was grown overnight in TSB, harvested by centrifugation, washed and resuspended in phosphate buffered saline (PBS) or PBS supplemented with protein (hemoglobin, myoglobin, or apohemoglobin (5 mg/ml)) or/and anhydrotetracycline (200 ng/ml) as indicated. Cotton rats were anesthetized, and a 10-µl aliquot containing 1×10^8^ or 1×10^5^ colony forming units (CFUs) was intranasally instilled drop-wise equally between the two nostrils. After 5 days the animals were sacrificed and the noses were surgically removed. The noses were placed in 1 ml of PBS containing 0.5% Tween-20, homogenized, and dilution plated onto TSA supplemented with 7.5% NaCl and spectinomycin (200 µg/ml) to determine CFUs. All animal experiments were conducted in strict accordance with the recommendations in the Guide for the Care and Use of Laboratory Animals of the National Institutes of Health. The protocol was approved by the University of Michigan University Committee on Use and Care of Animals (UCUCA) approval number 10394.

Nasal secretions were collected from a convenience sample of 20 adult patients in a rhiniology clinic within the University of Michigan Department of Otolaryngology and 10 healthy volunteers not visiting a clinic (University of Michigan Institutional Review Board approval number IRB00001996). Written informed consent was provided by study participants. Clinic patients were seen for evaluation of a variety of concerns, including nosebleeds, nasal blockage, rhinitis, and sinusitis. Nasal examination was performed as part of the routine clinical evaluation. For experimental purposes, the anterior nare was swabbed and the swab was streaked onto Mannitol Salt Agar to determine colonization by *S. aureus*. Secretions were collected by thoroughly swabbing the anterior and posterior nasal passageways with a sterile cotton swab (Remel BactiSwab). The tip of the swab was cut from the shaft, placed in a ridged eppendorf tube and centrifuged to obtain secretions. Volumes of secretions obtained varied from ∼50 µl to ∼200 µl. Secretions were stored at −20°C. Collected secretions were vortexed and sonicated to break up clumps then passed through a 0.22 µm syringe filter. To determine major protein composition of the secretions, 15 µl of samples were separated by 10% sodium dodecyl sulfate (SDS)-PAGE and stained with Sypro Ruby (Biorad). Visible bands were identified by in gel trypsin digestion and subsequent LC-MS/MS analysis (MS Bioworks, Ann Arbor, MI). Statistical analysis was utilized to determine the major protein component of each excised band and the major protein constituents were used for further experiments. The value for the abundance measurement is the Normalized Spectral Abundance Factor (NSAF) [26]. Western blots to detect the presence of hemoglobin in nasal secretions were done using the primary antibody anti-Human Hemoglobin whole antiserum produced in rabbit (Sigma H4890) at a dilution of 1∶100 in 5% milk. The secondary antibody was anti-rabbit IgG produced in goat, conjugated to peroxidase (Sigma A0545), used at a concentration of 1∶10,000 diluted in 5% milk.

### Biofilm experiments and adherence assays

Microtiter plate biofilms and flow cell biofilms were grown as previously described [27]. The growth medium for microtiter biofilms was 66% TSB supplemented with 0.2% glucose or with 10% (volume) nasal secretion or indicated concentration of protein. Microtiter plates used for this assay were cell culture treated 96 well (Costar 3596) or 384 well (Nunc 164688) plates. Flow cell biofilms were grown in 2% TSB with 0.2% glucose or 5 µg/ml hemoglobin. Confocal scanning laser microscopy and image analysis was performed as described previously [27]. Adherence assays to collagen and fibronectin were performed as previously described [28], [29]. Briefly, 96-well plates (Nunc 265301) were coated with the following proteins in PBS: Type IV collagen from human placenta (20 µg/ml; Sigma), fibronectin from human plasma (1 µg/ml; Sigma), or Bovine Serum Albumin (10 mg/ml; Fisher). 100 µl of protein was added to each well and incubated overnight at 4° with constant agitation. The wells were then washed three times with 1% BSA in PBS for 20 minutes at 37° with agitation. *S. aureus* was grown overnight in Brain Heart Infusion with 50 µg/ml of Human Hemoglobin. Following a one hour incubation at 37°C with agitation, wells were washed three times with PBS to remove non-adherent bacteria, then fixed with 2.5% glutaraldehyde in PBS for two hours at 4°C. Bacteria were stained with .1% crystal violet for 30 minutes at room temperature then washed three times with water. The stain was extracted by incubation with .2% Triton X-100 for 30 minutes at room temperature. The absorbance at 570 nm was read on a microtiter plate reader (Tecan Infinite M200).

### RNAIII expression assays

To monitor expression from the RNAIII promoter (P3), an overnight culture of the appropriate reporter strain was inoculated into TSB with Cam and grown to an optical density at 600 nm of 0.05. In triplicate, 475 µl of reporter culture was aliquoted into test tubes and indicated amounts of hemoglobin (or other protein) were added. The tubes were shaken at 250 rpm at 37°C for 12 hours. Both cell density (optical density at 595 nm) and green fluorescent protein (GFP) fluorescence (excitation at 485 nm, emission at 535 nm) were measured in a Tecan Infinite M200 (Research Triangle Park, NC) microtiter plate reader by removing 100 µl from each tube and assaying in a microtiter plate (Corning 3606).

### Autoinducing peptide activation assays with hemoglobin

*S. aureus* type 1 autoinducing peptide (AIP) was prepared as previously described using strain AH426 [27]. Briefly, an overnight preculture of expression strain AH426 was prepared and inoculated into 100 ml of Luria-Bertani broth with Amp. The culture was grown at 37°C with shaking until an optical density at 600 nm of 0.5 was reached, and IPTG (isopropyl-ß-d-thiogalactopyranoside) was added to a 0.5 mM final concentration. The culture was grown with shaking at 30°C for 3 h, and the cell pellets were stored at −70°C. Cell pellets were resuspended in 20 ml chitin binding buffer consisting of 100 mM phosphate buffer, pH 7.0, with 500 mM NaCl, 1 mM EDTA, 150 µl protease inhibitor cocktail (Sigma; catalog number P8465), and 0.5 mM phenylmethylsulfonyl fluoride. The cell suspension was lysed through two passes in a French press, and insoluble material was removed by centrifugation at 19,000 rpm for 30 min at 4°C in a Beckman JA-20 rotor. The supernatant was removed, 4 ml equilibrated 50% chitin beads (New England Biolabs) was added, and the resin suspension was mixed gently at room temperature for 30 min. The chitin resin was removed by centrifugation at 500×*g* for 5 min. The supernatant was removed, and the resin was washed three times for 5 min with 25 volumes of chitin binding buffer. The resin suspension was poured into a 10-ml column and allowed to settle by gravity (2-ml final resin volume), and the resin was equilibrated with three column volumes of elution buffer [100 mM phosphate, pH 7, 50 mM NaCl, 1 mM EDTA, 1 mM tris(2-carboxyethyl)phosphine (TCEP)]. Gravity flow from the column was stopped, and the resin was left sealed at room temperature for 15 h. Following incubation, fractions were eluted and assayed for activity or saved at −20°C. To determine AIP concentration the following was done: A Sep-Pak Plus cartridge (Waters, Milford, MA) was conditioned according to the manufacturer's instructions. To remove TCEP, an AIP sample from an intein purification was bound to the cartridge, washed with 20 ml of water with 0.1% trifluoroacetic acid (TFA), and eluted with 2 ml of 60% acetonitrile with 0.1% TFA. The concentration of the AIP was determined using assays with 5,5′-dithio-bis-(2-nitrobenzoic acid) (DTNB), also called Ellman's reagent (Pierce, Rockford, IL). The thiolactone ring was opened with 1 M (final concentration) NaOH and neutralized with HCl, and DTNB assays were performed before and after base treatment. For the assays, a 1-ml reaction mixture was prepared with 100 mM Tris-HCl, pH 8, and 0.1 mM DTNB (prepared fresh) and different amounts of untreated and base-treated AIP were added. The reaction mixtures were incubated for 10 min at room temperature, and the absorbance was measure at 412 nm. The concentrations were determined with an extinction coefficient of 13,600 M^−1^ cm^−1^, and the prebase reading was subtracted to get the final AIP concentration.

AIP activation assays were performed by incubating 100 nM of AIP with a human hemoglobin solution (5 µg/ml in PBS) for 2 hours at 30°C. Hemoglobin was removed from indicated samples and nasal secretions by adding 2 µl anti-hemoglobin antibody produced in rabbit (Sigma) with a 1 hour incubation, followed by addition of 5 µg of goat anti-rabbit IgG Magnetic Beads (New England Biolabs) with a 1 hour incubation at room temperature with periodic rocking. Tubes were then placed in a magnetic tube separation rack for 30 minutes, after which time the supernatant was collected. The AIP activation assay was performed by growing overnight cultures of *S. aureus* reporter strain (AH462) in TSB and subculturing 1∶50 into TSB plus 0.2% glucose supplemented with AIP; AIP+hemoglobin; or AIP pretreated with hemoglobin that was removed by antibody pulldown as described above. Cultures were grown in test tubes at 37°C with shaking at 250 rpm. Cell density and fluorescence were monitored at 8 hours after inoculation.

### Statistical analyses

Statistics were performed using a 1-way analysis of variance (ANOVA). Results are expressed as mean ± standard error of the mean, unless otherwise indicated.

## Results

### Nasal secretions from some *S. aureus* carriers have the ability to promote *S. aureus* surface colonization

**Figure 1:**
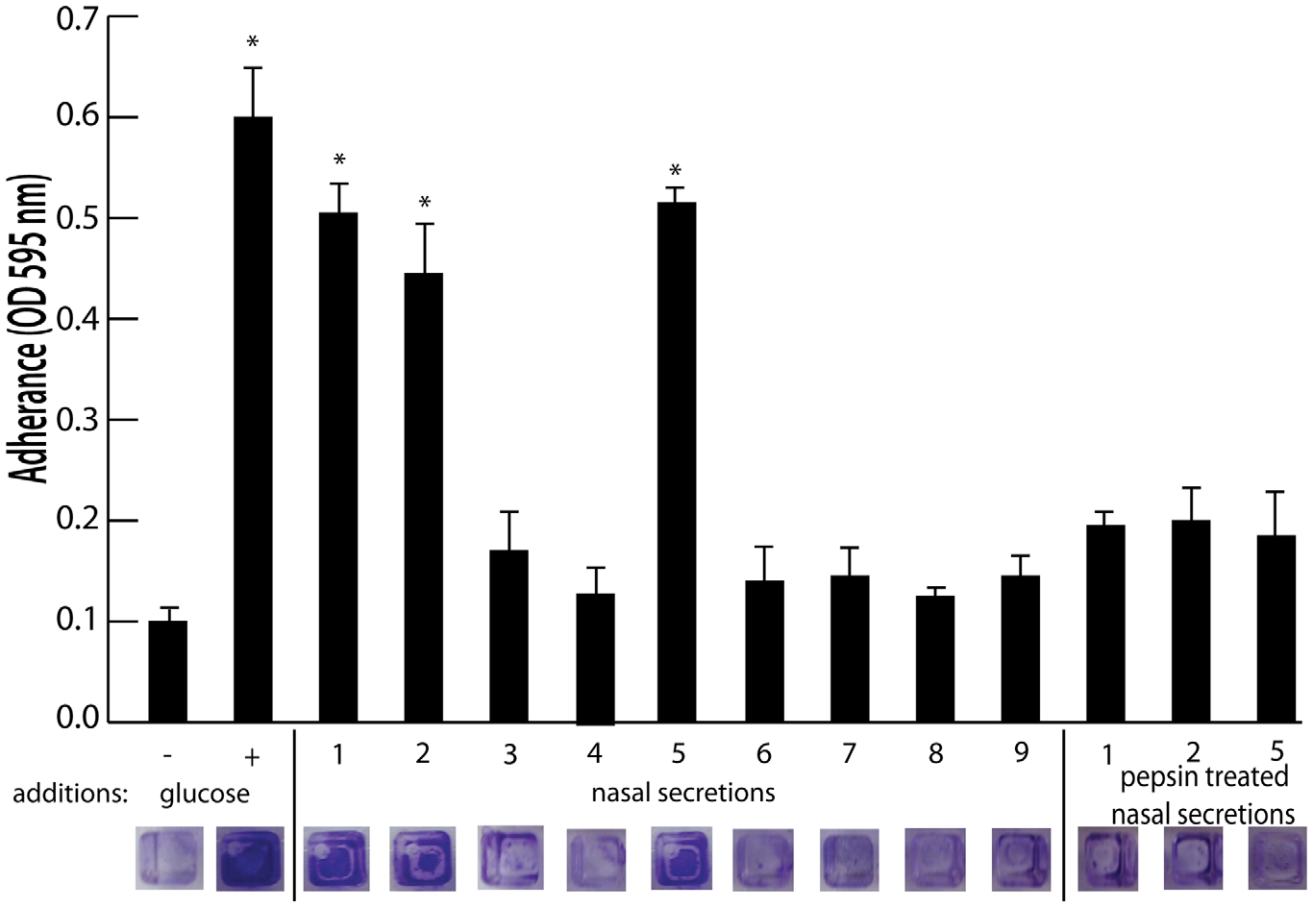
Effect of nasal secretions on *S. aureus* surface colonization. Shown is surface colonization in a 384 well plate with: 66% tryptic soy broth with or with out 0.2% glucose (columns 1 and 2); 66% tryptic soy broth supplemented with 10% filter sterilized nasal secretions from nine human volunteers (columns 3–11); and 66% tryptic soy broth supplemented with pepsin digested nasal secretions (secretions 1,2, and 5; columns 12–14). Graph shows quantitation of surface attached biomass and images below are crystal violet stained biomass attached to wells of a 384 well microtiter plate. Error bars show standard error of the mean; * P<0.005 versus unsupplemented tryptic soy broth (column 1).

**Figure 2:**
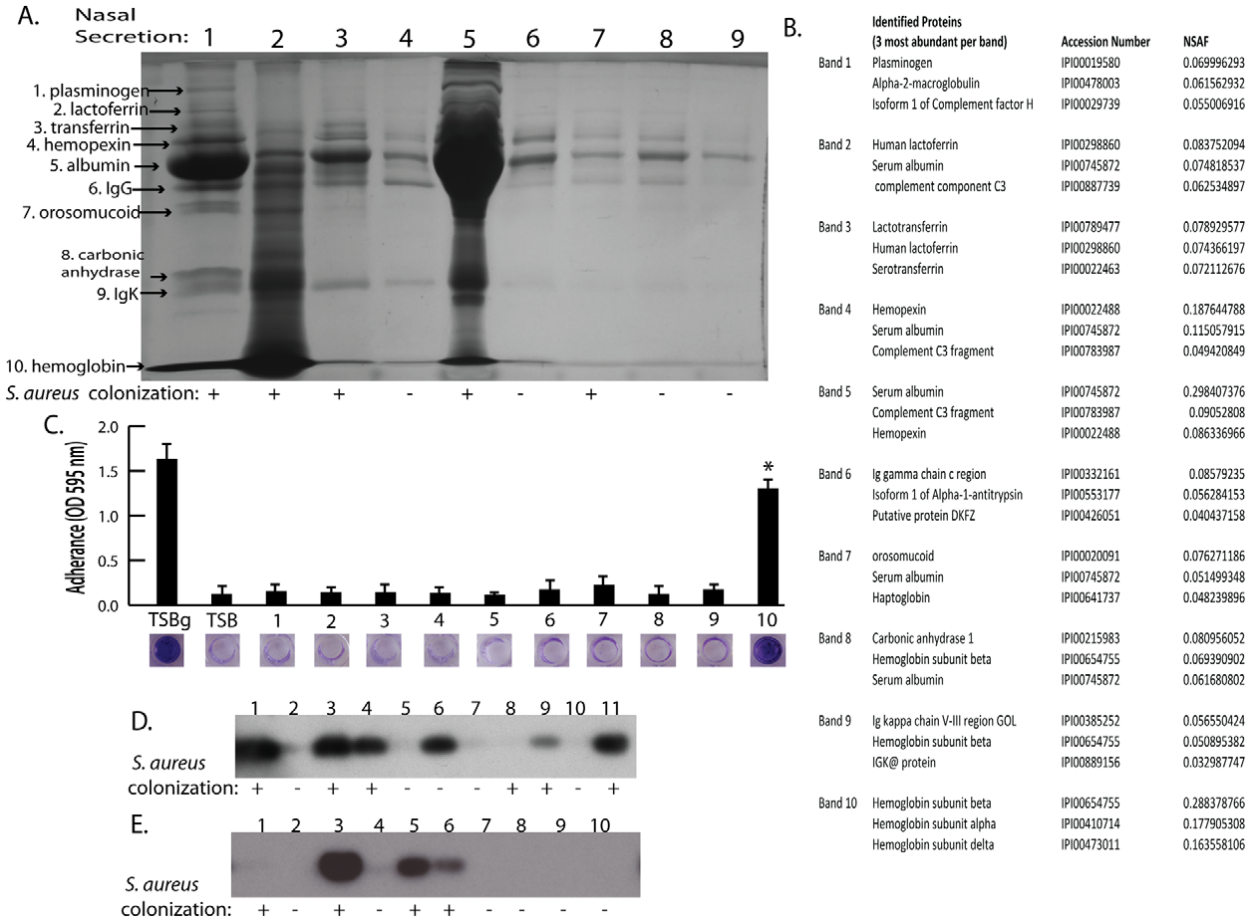
Nasal secretion proteins and their ability to promote *S. aureus* surface colonization. (A) SDS-PAGE analysis on 15 microliters of nasal secretions from the nine human volunteers (lanes 1–9). Protein names and arrows on left indicate protein identity determined by LC-MS/MS. *S. aureus* colonization status of each individual is indicated below gel. (B) Data set for the MS-based protein identifications from nasal secretions. The three most abundant proteins detected from each excised band are shown. (C) *S. aureus* surface colonization assay in the presence of individual proteins identified in nasal secretions. Columns 1 and 2 are positive and negative controls grown 66% TSB with (TSBg) or without (TSB) 0.2% glucose. Columns 3–12 were grown in 66% TSB supplemented with 10 µg/ml of each individual protein found in nasal secretions, 1: plasminogen, 2: lactoferrin, 3: transferring, 4: hemopexin, 5: albumin, 6: IgG, 7: orosomucoid, 8: carbonic anhydrase, 9: IgK, 10: hemoglobin. Graph shows quantitation of surface attached biomass and images below are crystal violet stained biomass attached to wells of a 96 well microtiter plate. Error bars show standard error of the mean; * P<0.001 versus unsupplemented tryptic soy broth. (D and E) Western blot analysis on nasal secretions using anti-hemoglobin antibody to detect the presence or absence of hemoglobin. (D) is a western blot on 11 nasal secretions from 11 clinic patient volunteers distinct from those in Figure 2A. (E) is a western blot on 10 nasal secretions from 10 healthy volunteers. *S. aureus* nasal colonization status is indicated below each blot.

To identify host factors that contribute to *S. aureus* nasal carriage, we collected nasal secretions from patients at the University of Michigan Otolaryngology Clinic and analyzed them for the ability to promote *S. aureus* surface colonization. Tryptic soy broth was supplemented with filtered nasal secretions (10% of total volume) from 9 subjects and analyzed for the ability to promote *S. aureus* surface colonization in triplicate wells of a 384 well plate. 384 well plates were utilized for this assay to reduce the amount of volume necessary, as a limited volume of secretion (∼50–200 µL) was collected from each subject. Secretions 1, 2, and 5 significantly increased *S. aureus* surface colonization (Figure 1). Protease treatment of secretions 1, 2, and 5 with pepsin-coated beads resulted in the loss of ability to promote surface colonization, suggesting a protein component was responsible for induction of surface colonization. Nasal swabs taken at the time of secretion collection revealed that individuals 1, 2, 3, 5, and 7 were colonized with *S. aureus* (Figure 2A).

### Hemoglobin can promote *S. aureus* surface colonization

The nasal secretions were next examined by sodium dodecyl sulfate polyacrylamide gel electrophoresis (SDS-PAGE) and all visible protein bands from secretion 1 were identified by mass spectrometry (Figure 2A). Secretion 1 contained plasminogen, lactoferrin, transferrin, hemopexin, albumin, IgG, orosomucoid, carbonic anhydrase, IgK, and hemoglobin (alpha and beta subunits) (Figure 2B). Next, these proteins were analyzed individually to determine if any were capable of promoting *S. aureus* surface colonization (Figure 2C). We found that purified hemoglobin promoted *S. aureus* surface colonization. None of the other proteins tested promoted *S. aureus* surface colonization.

**Figure 3:**
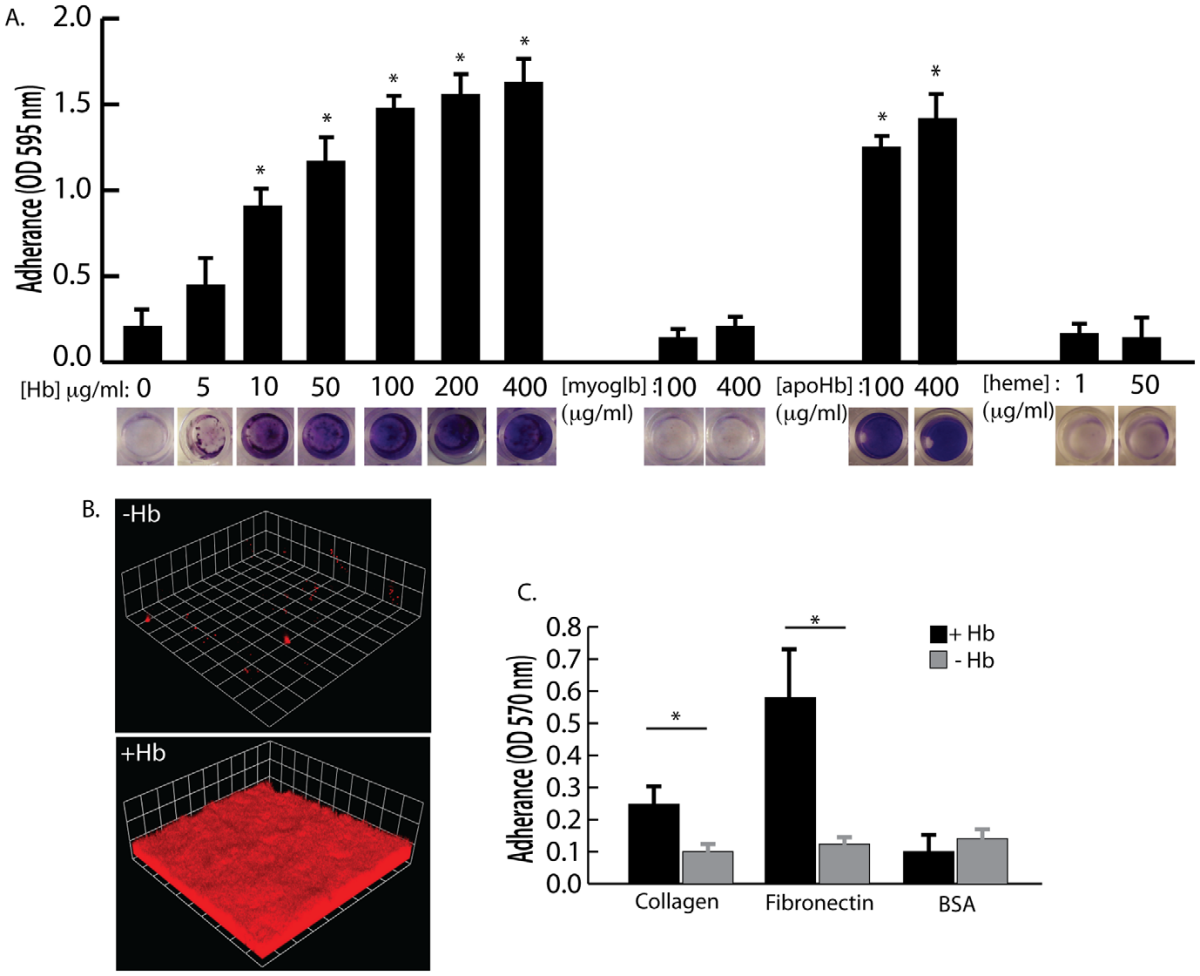
The effect of hemoglobin on *S. aureus* surface colonization. (A) *S. aureus* surface colonization in 66% TSB supplemented with human hemoglobin, myoglobin, apohemoglobin, or heme (concentrations indicated in graph). Graph shows quantitation of surface attached biomass and images below are crystal violet stained biomass attached to wells of a 96 well microtiter plate. Error bars show standard error of the mean; * P<0.005 versus unsupplemented tryptic soy broth (B) Confocal laser scanning microscopy (CLSM) of *S. aureus* grown in flow cells with tryptic soy broth without (−Hb) or with 5 µg/ml hemoglobin (+Hb). Shown are three dimensional image reconstructions of a z series created with Velocity software. CLSM images are representative of three separate experiments and each side of a grid square represents 10 micrometers. (C) Adherence assay of *S. aureus* grown in the presence or absence of 50 µg/ml hemoglobin to collagen and fibronectin. Error bars show standard error of the mean; * P<0.01.

Hemoglobin concentrations of 10 µg/ml and higher were found to significantly increase *S. aureus* surface colonization in microtiter plate assays and flow cell assays (Figure 3A and 3B). Other heme containing proteins, such as myoglobin, or heme itself did not possess the ability to promote *S. aureus* surface colonization (Figure 3A). In addition, apohemoglobin promoted surface colonization in the same concentration range as hemoglobin, suggesting iron is not the cause of this phenomenon.

Adherence assays were also performed to determine if growth in the presence of hemoglobin could influence initial attachment to the human extracellular matrix proteins collagen and fibronectin (Figure 3C). *S. aureus* grown in the presence of hemoglobin attached to both collagen and fibronectin at significantly higher levels than *S. aureus* grown in the absence of hemoglobin. These results are consistent with previous reports suggesting that growth on blood agar plates is ideal for expression of surface binding proteins by *S. aureus* [29], [30].

**Figure 4:**
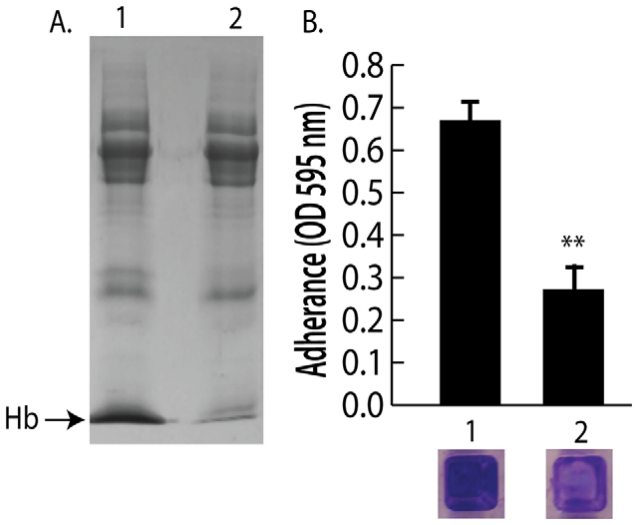
Depletion of hemoglobin from a nasal secretion results in reduced *S. aureus* surface colonization. (A) A single hemoglobin containing secretion collected from one volunteer was split in half; one half was untreated and the other half was depleted of hemoglobin by immunoprecipitation (A) lane 1 (untreated) versus lane 2 (hemoglobin IP). (B) Promotion of surface colonization by nasal secretion shown in (A); 1 is untreated and 2 is hemoglobin depleted secretion. Error bars show standard error of the mean; ** P<0.01 versus Hb containing secretion (column 1).

Because nasal secretions are complex mixtures of protein, sugars, and salts, hemoglobin was specifically depleted by immunoprecipitation to test the hypothesis that hemoglobin was a necessary factor in inducing bacterial surface colonization. Additional nasal secretions were collected and one that contained hemoglobin and promoted *S. aureus* surface colonization was processed as described above to remove clumps and debris. Half of this secretion was left untreated while the other half was incubated with anti-hemoglobin antibody that was then immunoprecipitated with a secondary antibody conjugated to magnetic beads. Figure 4A shows SDS-PAGE analysis of the untreated (lane 1) and hemoglobin immunoprecipitated nasal secretion (lane 2). Immunoprecipitation of hemoglobin significantly reduced the ability of this nasal secretion to promote *S. aureus* surface colonization (Figure 4B). Of note, one protein band that was not hemoglobin nonspecifically immunoprecipitated in this experiment so we cannot rule out the possibility that this unidentified protein could have an impact on the colonization assay.

We next collected nasal secretions and performed nasal swabs on 11 additional individuals visiting the clinic and 10 healthy individuals to determine if a correlation between the presence of hemoglobin in nasal secretions and *S. aureus* colonization persist in an additional sampling (Figure 2D and 2E). Six of the secretions from clinic patients (Figure 2D lanes 1,3,4,6,9,11) had detectable hemoglobin by western blotting and five of these secretions came from individuals determined to be nasally colonized with *S. aureus*. Analysis of healthy volunteers revealed detectable hemoglobin in nasal secretions from three individuals and all three of these volunteers were nasally colonized with *S. aureus* (Figure 2E lanes 3,5,6).

### The presence of hemoglobin promotes *S. aureus* nasal colonization

**Figure 5:**
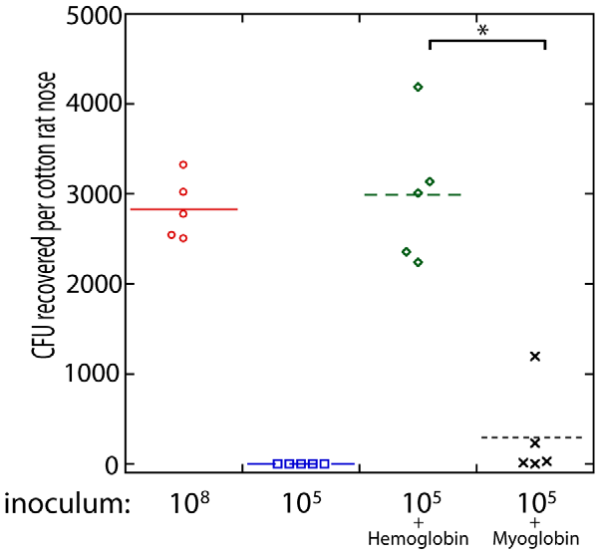
Nasal colonization of *S. aureus* in the cotton rat model. The inoculum (in colony forming units) used to initially colonize the rat nose is shown below the graph. Bacteria were suspended in phosphate buffered saline (PBS) or PBS with hemoglobin (5 mg/ml), myoglobin (5 mg/ml), or apohemoglobin (5 mg/ml) as indicated. Bacterial colony forming units in the cotton rat nose 5 days after instillation are shown in the graph. Medians are given as horizontal lines. * P<0.001, ** P<0.01.

To determine if the presence of hemoglobin could influence *S. aureus* nasal colonization, we utilized the cotton rat model [25]. Nasal instillation with 10 µl of a *S. aureus* suspension at a density of 1×10^8^ colony forming units resulted in reproducible colonization after 5 days (Figure 5). A reduced inoculum, 10 µl of a *S. aureus* suspension at a density of 1×10^5^ colony forming units, resulted in no isolation of nasal *S. aureus* after 5 days. However, if the reduced inoculum (1×10^5^) was suspended in a solution of hemoglobin at 5 mg/ml, nasal colonization was reproducibly observed 5 days after instillation. This high concentration of hemoglobin was utilized in an attempt to keep hemoglobin present in the nasal passageway for as long as possible. Myoglobin supplementation of the reduced inoculum (1×10^5^) did not significantly increase *S. aureus* nasal colonization. Supplementation with apohemoglobin resulted in robust nasal colonization similar to the hemoglobin condition. These results suggest that the presence of hemoglobin in nasal secretions, but not all heme containing proteins, can increase the likelihood of *S. aureus* nasal colonization given a small inoculum.

### Hemoglobin inhibits expression of the *agr* quorum sensing system

**Figure 6:**
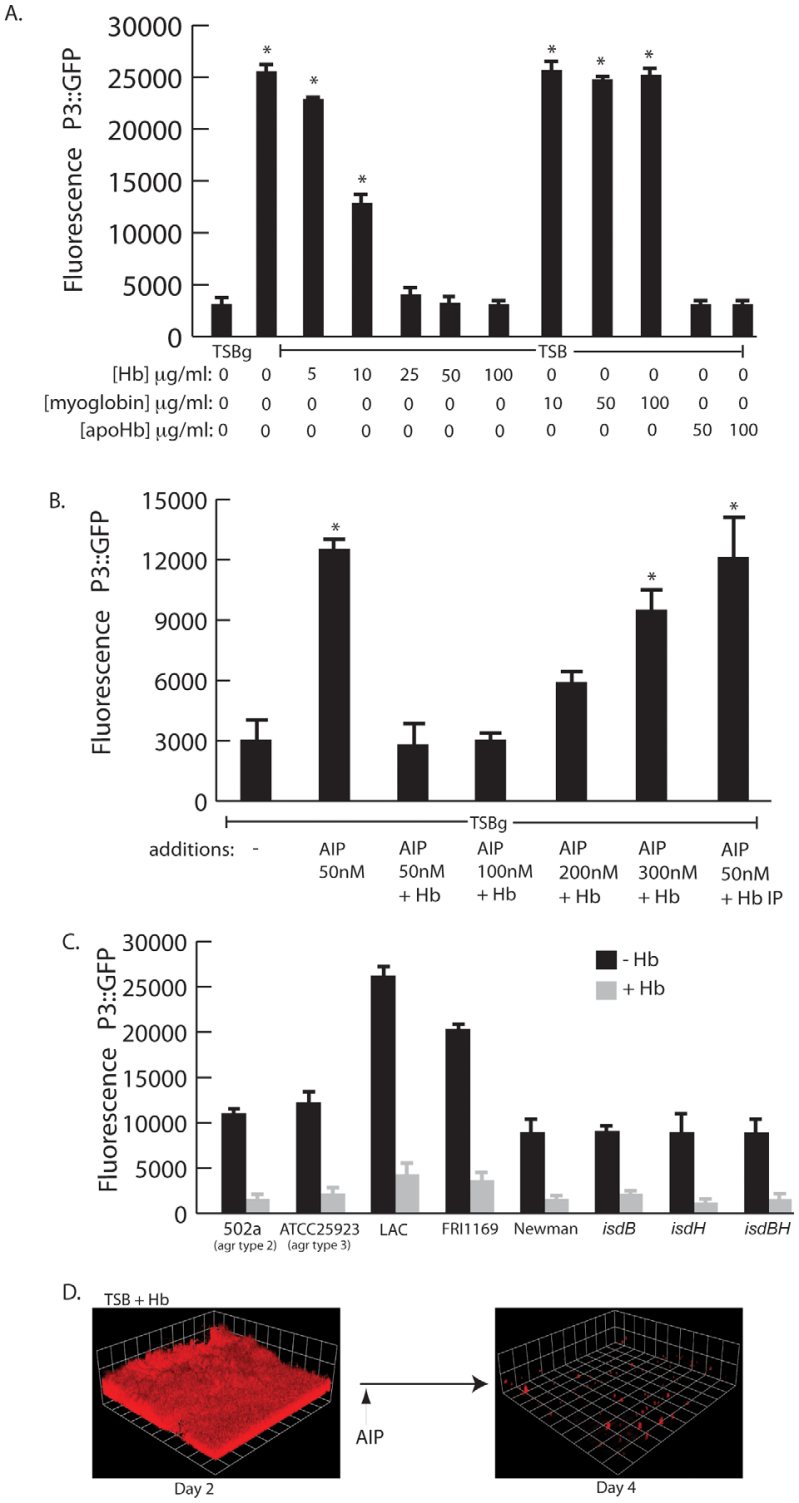
*agr* activity in the presence of hemoglobin. (A) Measurement of agr P3::GFP reporter activity in wildtype *S. aureus* (strain AH462) grown for 12 hours in TSBg or TSB supplemented with indicated concentrations of hemoglobin, myoglobin, or apohemoglobin. Error bars show standard error of the mean; * P<0.001 versus TSBg condition. (B) *agr* activation assay in the presence of hemoglobin. Reporter strain (AH462) was grown in 66% TSB with 0.2% glucose. At the beginning of logarithmic phase the cultures were supplemented with: 50 nM AIP, 50–300 nM AIP+5 ug/ml hemoglobin, or 50 nM AIP preincubated with hemoglobin that was subsequently immunoprecipitated. Error bars show standard error of the mean; * P<0.01 versus no addition control. (C) Effect of hemoglobin on agr activation on different *agr* types (502a – type 2, ATCC25933 –type 3), multiple *S. aureus agr* type 1 strains (LAC, FRI1164, Newman) and *isd* mutants. Error bars show standard deviation. (D) AIP induced biofilm detachment in the presence of hemoglobin. Biofilms were grown for 48 hours in TSB supplemented with 2.5 µg/ml of hemoglobin. After 48 hours, 2 ml of 25 µM AIP was added into the biofilm growth media.

Biofilm formation and lack of biofilm dispersal often correlate with reduced expression of the *agr* quorum sensing system [27], [31], [32], [33]. Induction of the *agr* system results in the increased production of several secreted virulence factors including proteases, hemolysins, and toxins [34], [35]. Because a recent report by Schlievert *et. al.* [36] demonstrated that hemoglobin found in menses inhibits production of secreted exotoxins, we hypothesized that hemoglobin would inhibit *agr* expression. To determine if hemoglobin was affecting *agr* expression we followed expression of the quorum sensing responsive promoter fusion, P3-GFP, in the presence of increasing concentrations of hemoglobin (Figure 6A). Hemoglobin significantly inhibited expression from the P3 promoter measured after 12 hours of growth at concentrations from 10–100 µg/ml. Control experiments with myoglobin did not result in reduced expression from the P3 promoter but supplementation with apohemoglobin inhibited P3 expression, suggesting the activity is specific to the hemoglobin peptide rather than any heme containing protein.

We reasoned that hemoglobin could antagonize activation of the *agr* P3 promoter either by sequestering autoinducing peptide (AIP) or directly binding to the cell surface and interfering with the *agr* activation process. To delineate between these two possibilities we incubated purified AIP with hemoglobin, removed the hemoglobin by immunoprecipitation, and assayed the ability of the remaining solution to activate expression from the P3 promoter (Figure 6B). A mixture of hemoglobin and AIP at a concentration of 50 nM was unable to activate expression of the P3 promoter compared to AIP alone. However, when AIP concentration levels were increased this inhibition was overcome. If hemoglobin was depleted by immunoprecipitation from a mixture containing 50 nM AIP the remaining solution possessed P3 activation activity, suggesting that hemoglobin does not sequester AIP. In addition, *S. aureus* biofilms grown in the presence of hemoglobin dispersed upon addition of AIP (Figure 6D).

There are four types of *agr* quorum sensing systems among *S. aureus* strains. Each *agr* system (*agr-I* through *agr-IV*) recognizes a unique autoinducing peptide structure (AIP-1 through AIP-4). These *agr* types can be divided into three cross-inhibitory groups: *agr-*I/IV, *agr-*II, and *agr-*III. Hemoglobin was found to inhibit induction of the P3 promoter in each *agr* class and in multiple strains (Figure 6C). We also examined mutants in receptors known to bind hemoglobin (*isdB* and *isdH*) [37] and found no difference in the ability of hemoglobin to inhibit *agr* quorum sensing this these genetic backgrounds. These results suggest the ability of hemoglobin to inhibit quorum sensing is spread across all *agr* types and does not occur by binding of the AIP peptide or via binding to known hemoglobin receptors (IsdB or IsdH).

### Constitutive expression of the *agr* quorum sensing effector molecule, RNAIII, inhibits *S. aureus* nasal colonization

**Figure 7:**
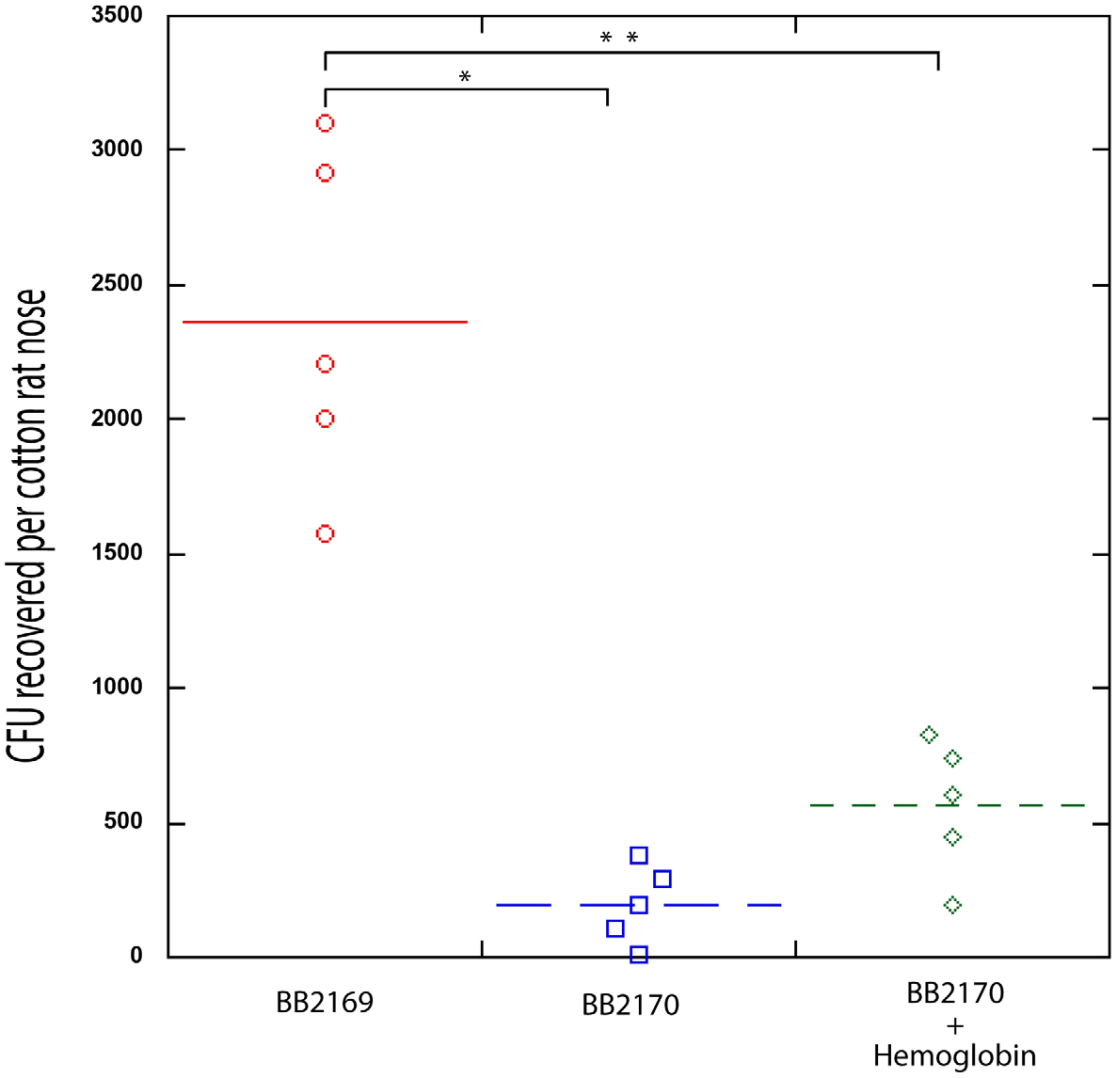
Nasal Colonization of *S. aureus* containing a *RNAIII* expression vector (BB2170) or vector control (BB2169) in the cotton rat model. Bacterial colony forming units in the cotton rat nose 5 days after instillation are shown in the graph. Medians are given as horizontal lines. *P<0.0001; ** P<0.001.

Recent work by Burian *et. al.* has shown that expression of the *agr* quorum sensing system is minimal during nasal colonization [38], [39]. Based on this finding and the knowledge that hemoglobin can promote *S. aureus* nasal colonization and inhibit the expression of the *agr* quorum sensing system, we hypothesized that expression of RNAIII, the *agr* quorum sensing system effector molecule, would reduce nasal colonization. To test this hypothesis we utilized a plasmid containing promoterless RNAIII cloned behind a tetracycline inducible promoter, pALC2073-RNAIII. A similar construct has previously been shown to reduce expression of surface associated adhesins and reduce biofilm formation in the presence of tetracycline [40]. In the rat nasal colonization model, presence of pALC2073-RNAIII resulted in reduced nasal colonization, even in the presence of hemoglobin (Figure 7). These results suggest that repression of the *agr* quorum sensing system is necessary for efficient *S. aureus* nasal colonization and factors capable of inhibiting *agr* expression may promote nasal colonization.

## Discussion

Nasal carriage of *S. aureus* is a major risk factor for developing a range of infections in both clinical and community settings [6]–[9]. Nasal colonization is multifactorial, likely involving both host and bacterial determinants [11]–[21], [41], [42]. Current strategies for eradication of *S. aureus* nasal carriage include the use of topical mupirocin; however mupirocin resistance is appearing suggesting that susceptibility may not be long lasting [43], [44]. Therefore, further advances in the control of *S. aureus* colonization are needed and will depend on an in-depth understanding of both host and bacterial determinants of carriage. In this work, we describe a host factor, hemoglobin, and a bacterial molecular mechanism, expression of the *agr* quorum sensing system, that influence *S. aureus* nasal colonization.

The collection and analysis of human nasal secretions from nine volunteers revealed that secretions from three subjects had the ability to significantly promote *S. aureus* surface colonization (Figure 1). Protease digestion of these secretions eliminated their ability to promote surface colonization, suggesting the involvement of proteinaceous factors. Major protein components of one of the secretions were identified and individual analysis of each protein revealed that hemoglobin possessed the ability to promote *S. aureus* surface colonization (Figures 2 and 3). Depletion of hemoglobin from a nasal secretion resulted in reduced *S. aureus* surface colonization (Figure 4). Furthermore, in a rat model the presence of hemoglobin reduced the size of the inoculum necessary to achieve nasal colonization (Figure 5). Finally, we found that hemoglobin prevented expression of the *agr* quorum sensing system (Figure 6) and the aberrant constitutive expression of RNAIII resulted in reduced nasal colonization (Figure 7). Collectively these results suggest that hemoglobin is a host factor whose presence in nasal secretions contributes to *S. aureus* nasal colonization.

Several *S. aureus* factors have been described as determinants for nasal colonization, including: wall teichoic acid, the iron-regulated surface determinant A, catalase, alkyl hydroperoxide reductase, clumping factor B, sortase A, and the autolysin SceD [15]–[21]. Here we show that the aberrant constitutive expression of RNAIII, the *agr* quorum sensing effector molecule, results in reduced nasal colonization. This finding correlates well with recent data that *agr* is not expressed during nasal colonization in the cotton rat model or in humans [38], [39]. At this time it is unclear which *agr* regulated factors influence nasal colonization. Recent work has demonstrated that the *Staphylococcus epidermidis* secreted protease Esp is capable of preventing and eradicating *S. aureus* nasal colonization [22]. Considering that the expression of several secreted proteases are up-regulated by the *agr* regulatory system and the expression of *agr*-regulated proteases results in biofilm dispersal [27], [35], we speculate that *S. aureus* protease expression may result in reduced nasal colonization. Further work is needed to elucidate the role of *agr*-regulated factors in nasal colonization.

The mechanism by which hemoglobin influences *S. aureus agr* expression and nasal colonization is not clear. Hemoglobin is an iron (heme) containing protein, but other iron or heme containing proteins did not produce the same effect and apohemoglobin elicited the same responses as hemoglobin. Our data suggest that hemoglobin is not able to bind and sequester autoinducing peptides, so hemoglobin likely acts by binding to the cell surface. Mutations of known hemoglobin binding proteins, IsdB and IsdH, exhibited no difference in their response to hemoglobin, suggesting they are not involved in this phenomenon. Agr inhibition also occurs in multiple strains and *agr* types. Hemoglobin has regions of high positive charge that could promote interaction with negatively charged phospholipids and interfere with activation of membrane histidine kinases such as AgrC. Indeed, work by Scheilvert *et al.* demonstrated that exotoxin regulation via the SrrA-SrrB two component system was affected by the presence of hemoglobin [36].

Our findings are consistent with a recent study that found individuals experiencing epistaxis (nose bleeds) are more likely to be nasally colonized with *S. aureus* than control individuals [45]. Some nasal secretions used in our study were collected from patients experiencing epistaxis, nasal blockage, rhinitis, or sinusitis, which presumably increased the likelihood of hemoglobin being present in their secretions. However microepistaxis is thought to be common and can result from digital trauma (*i.e.*, nose picking), nasal or sinus infections, dry ambient air, and from the use of topical nasal medications such as antihistamines and corticosteroids [46], [47]. Sampling of nasal secretions from 10 healthy individuals revealed the presence of hemoglobin in secretions from 3 individuals, all of whom were nasally colonized by *S. aureus*. Therefore it seems possible that hemoglobin is commonly present in nasal secretions and we propose this could contribute to *S. aureus* nasal colonization. A large sampling of healthy individuals is needed to determine if there is a significant correlation between *S. aureus* nasal colonization and the presence of hemoglobin in nasal secretions.

The presence of hemoglobin may also play an important role in *S. aureus* colonization of other body sites. Recent work has demonstrated that staphylococcal exotoxins were not produced when the organism was cultured in human menses and hemoglobin was identified as the inhibitory factor [36]. It seems plausible that hemoglobin present in vaginal secretions and menses could have profound effects on *S. aureus* vaginal colonization. Exposure to hemoglobin could also influence *S. aureus* bloodstream infections by limiting exotoxin production and promoting biofilm infections such as infective endocarditis.

Why would *S. aureus* have evolved to down regulate the *agr* quorum sensing system and colonize surfaces in the presence of hemoglobin? Since *S. aureus* preferentially uses hemoglobin as an iron source [48], it is likely advantageous to form a surface associated community where the scarce resource, iron, is available. Another non-exclusive possibility is that the production of some *agr* regulated secreted proteases could cleave hemoglobin, releasing antimicrobial peptides. Others have shown hemoglobin peptide fragments found in menses and ticks possess antimicrobial activity against *S. aureus* [49], [50]. Therefore by not producing *agr*-regulated proteases when hemoglobin is present, *S. aureus* may avoid the generation of these antimicrobials. In any case, *S. aureus* has evolved to respond to one of the most abundant proteins found in humans and this interaction could have profound effects on *S. aureus* colonization and pathogenesis.
